# Impact of an ultra-low dose unenhanced planning scan on CT coronary angiography scan length and effective radiation dose

**DOI:** 10.1259/bjro.20210056

**Published:** 2023-01-03

**Authors:** Laura Duerden, Helen O’Brien, Susan Doshi, Pia Charters, Laurence King, Benjamin. J Hudson, Jonathan Carl Luis Rodrigues

**Affiliations:** ^1^ Royal United Hospitals Bath NHS Foundation Trust, Avon, United Kingdom; ^2^ Velindre Cancer Centre, Velindre University NHS Trust, Cardiff, United Kingdom; ^3^ University of Bath, Bath, United Kingdom

## Abstract

**Objective:**

Imaged scan length (z-axis coverage) is a simple parameter that can reduce CT dose without compromising image quality. In CT coronary angiography (CTCA), z-axis coverage may be planned using non-contrast calcium score scan (CaCS) to identify the relevant coronary anatomy. However, standardised Agatston CaCS is acquired at 120 kV which adds a relatively high contribution to total study dose and CaCS is no longer routinely recommended in UK guidelines. We evaluate an ultra-low dose unenhanced planning scan on CTCA scan length and effective radiation dose.

**Methods:**

An ultra-low dose tin filter (Sn-filter) planning scan (100 kVp, maximum iterative reconstruction) was performed and used to plan the z-axis coverage on 48 consecutive CTCAs (62% men, 62 ± 13 years) compared with 47 CTCA planned using a localiser alone (46% men, 59 ± 12 years) between May and June 2019. Excess scanning beyond the ideal scan length was calculated for both groups. Estimations of radiation dose were also compared between the two groups.

**Results:**

Addition of an ultra-low dose unenhanced planning scan to CTCA protocol was associated with reduction in overscanning with no impact on image quality. There was no significant difference in total study effective dose with the addition of the planning scan, which had an average dose–length product of 3 mGy.cm. (total study dose: Protocol A 2.1 mSv *vs* Protocol B 2.2 mSv, *p* = 0.92).

**Conclusion:**

An ultra-low dose unenhanced planning scan facilitates optimal scan length for the diagnostic CTCA, reducing overscanning and preventing incomplete cardiac imaging with no significant dose penalty or impact on image quality.

**Advances in knowledge:**

An ultra-low dose CTCA planning is feasible and effective at optimising scan length.

## Background

CT coronary angiography (CTCA) is a widely used diagnostic imaging modality for assessing coronary artery disease (CAD). The high negative predictive value of CTCA means it can be used to effectively exclude significant CAD in patients who are low- to intermediate risk.^1^ The clinical impact has been demonstrated in the SCOT-HEART trial, where rapid access chest pain clinic patients were randomised to CTCA in addition to standard of care and had a significant reduction in death from coronary heart disease or non-fatal myocardial infarction at 5 year follow-up.^2^

For studies involving ionising radiation, the “as low as reasonably achievable” principle should apply. CT technique and operating factors need to be optimised to ensure radiation dose is as low as reasonably achievable, whilst maintaining diagnostic image quality.^3^ The radiation dose from CTCA studies varies widely across institutions and is dependent on many variables.^4^ There are several ways to reduce effective dose from CTCA investigations including prospective ECG triggered scanning, heart rate control, tube current modulation, optimised contrast monitoring, iterative reconstruction, and high pitch scanning.^5,6^

Imaged scan length (z-axis coverage) is a simple parameter that can be optimised without compromising image quality.^7,8^ This is particularly pertinent to helical acquisition in multidetector CT, where the irradiated scan length is longer than the imaged scan length, to enable image reconstruction. Extending the scan beyond the anatomical structures of interest constitutes unnecessary radiation dose to the patient, without adding relevant information of clinical utility. Conversely, scans that do not fully cover the region of interest and require subsequent phases also significantly increase total radiation dose and intravenous contrast dose of the study.

In CTCA, the ideal scan length extends from just cranial to the coronary tree to just caudal to the inferior-most aspect of the myocardium.^9^ Overscanning may increase the total scan length by approximately 10–15% for each additional centimetre included in the z-axis, as the heart has been shown to be around 10 cm in craniocaudal distance.^10^

Planning the z-axis coverage of CTCA can be done using a non-contrast planning scan, such as a calcium score scan, to identify the coronary arteries and cardiac anatomy and plan the required field of view.^9,11^ Calcium score scan (CaCS) may also be used to adjust other CTCA parameters such as gating and dose in order to optimise the CTCA study.^12^ Planning the z-axis coverage can also be done using anatomical landmarks on the localiser image(s), using the carina to define the cranial limit of the scan, and the cardiac apex as the caudal limit.^11^ The accuracy of this method is improved by performing both frontal and lateral localiser projections.^8^ However, the availability of two localiser projections is vendor-dependent and not available for all manufacturers. Leschka et al (2010)^11^ demonstrated that when CTCA is planned using localiser anatomical landmarks, the radiation dose of the CTCA is 16% higher than when the z-axis coverage is planned using a CaCS scan.

In the UK, it is no longer a standard practice to perform a calcium score for all patients and the recommended first-line imaging is CTCA.^13^ Whilst this may reduce total study dose, the opportunity to optimise the CTCA scan is lost. However, the relatively high contribution of CaCS to total study dose does not justify inclusion of a CaCS for CTCA planning alone.^14^ This is because a non-contrast calcium score scan using the Agatston method encompasses the whole heart and requires a relatively high tube potential of 120 kV. The radiation dose of a standard 120 kV CaCS scan is typically 1–3 mSv.^15^ CTCA is often performed at a lower tube potential with a radiation dose typically 2–8 mSv.^16^ Third generation dual source CT technology can achieve even lower doses, on average <1 mSv in one ‘real-world’ series.^17^ Jin et al (2020) demonstrated that planning CTCA coverage using the CaCS decreased the length of the CTCA and subsequent dose–length product (DLP), however the overall radiation dose was higher due to the relatively high dose contribution of a standard 120 kV CaCS.^18^

Third generation dual-source CT with Sn filtration can perform non-contrast scans with significantly less dose. The Sn filter absorbs lower energy X-rays around 30–50 keV which reduce the dose efficiency of non-contrast scans. This technology has recently been investigated for low kV coronary artery calcium scoring and has resulted reduction in radiation dose of around 75% compared to the 120 kVp acquisition required for standard Agatston calcium scoring,.^19,20^

As part of a dose reduction quality improvement cycle, we aimed to assess the impact of a low dose non-contrast planning scan on scan length and total examination dose for consecutive patients undergoing CTCA, compared to CTCA planned with a single frontal localiser. We hypothesised that the excess dose from the ultra-low dose non-contrast planning scan will be offset by dose saving associated with reduction in overscanning beyond the coronary arterial tree in routine CTCA.

## Methods

The study was part of a quality improvement cycle to reduce z-axis overscan in CTCA. Approval was obtained by the local institutional audit committee, confirming that full ethical approval and informed written consent were not required.

### Patient cohort

Two groups of consecutive patients who underwent CTCA as part of routine clinical practice were compared, before and after introduction of an ultra-low dose Sn-filter planning scan. Protocol A consisted of 47 consecutive patients who had undergone high-pitch FLASH mode CTCA, during a 1-month period in May 2019, who had CTCA only with z-axis coverage planned using a frontal localiser. Protocol B consisted of 48 consecutive patients who had undergone the ultra-low dose Sn-filter planning scan prior to high-speed high-pitch FLASH mode CTCA, during a 1-month period in July 2019. Data were collected retrospectively.

### CTCA technique and protocol

All FLASH mode CTCAs were performed on a 128 multidetector row CT system (Drive, Siemens Healthineers, Erlangen, Germany). All patients were examined in the supine position in the craniocaudal direction. Oral±intravenous beta blockade was used with a target heart rate of <60 bpm with beat-to-beat heart variability <3. Tube voltage and tube current reference values were 100kV (CARE kV) and 282 mAs (CARE dose). Collimation (128 × 0.6 mm), pitch (3.4) and rotation time (0.28) were equal between both groups. 60 ml of non-ionic intravenous contrast medium (Iohexol 350 mg Iodine/ml; Omnipaque 350, Amersham Health UK) was administered at 5 ml s^−1^ via a 16G cannula in the antecubital fossa. Automated verbal breathing instructions were given advising patients to ‘breathe in, breathe out, breathe in again and hold your breath’ before imaging.

In Protocol A, scan length was planned using the frontal localiser by a radiographer, under the supervision of a radiologist. Ideal image scan length was defined as extending from the carina to the cardiac apex.^11^

In Protocol B, FLASH CTCA was preceded by an additional ultra-low dose, non-contrast Sn-filter planning scan. Tube voltage and tube current reference values of the planning scan were Sn100 kV (Care kV) and 100 mAs (Care dose). The planning scan was reviewed by a radiologist to identify the coronary arteries, and this was used to set the cranial and caudal extent of the subsequent CTCA with as little excess z-axis coverage as possible.

For both groups, when repeat CTCA was required, *e.g.* in cases of motion artefact, the CTCA was repeated using a protocol at the discretion of the supervising radiologist.

### Measuring excess scan length

Sequential axial images were reconstructed from helical data and stored on a picture archiving and communication system for clinical interpretation (Fujifilm Synapse PACS v. 4, Tokyo, Japan). The images were viewed using standardised CT vascular window settings. The data sets were reconstructed into multiplanar reformatted images in the coronal and sagittal planes using cardiac specific post-processing software (Syngo.via, Siemens Healthineers, Erlangen, Germany).

**Figure 1.**
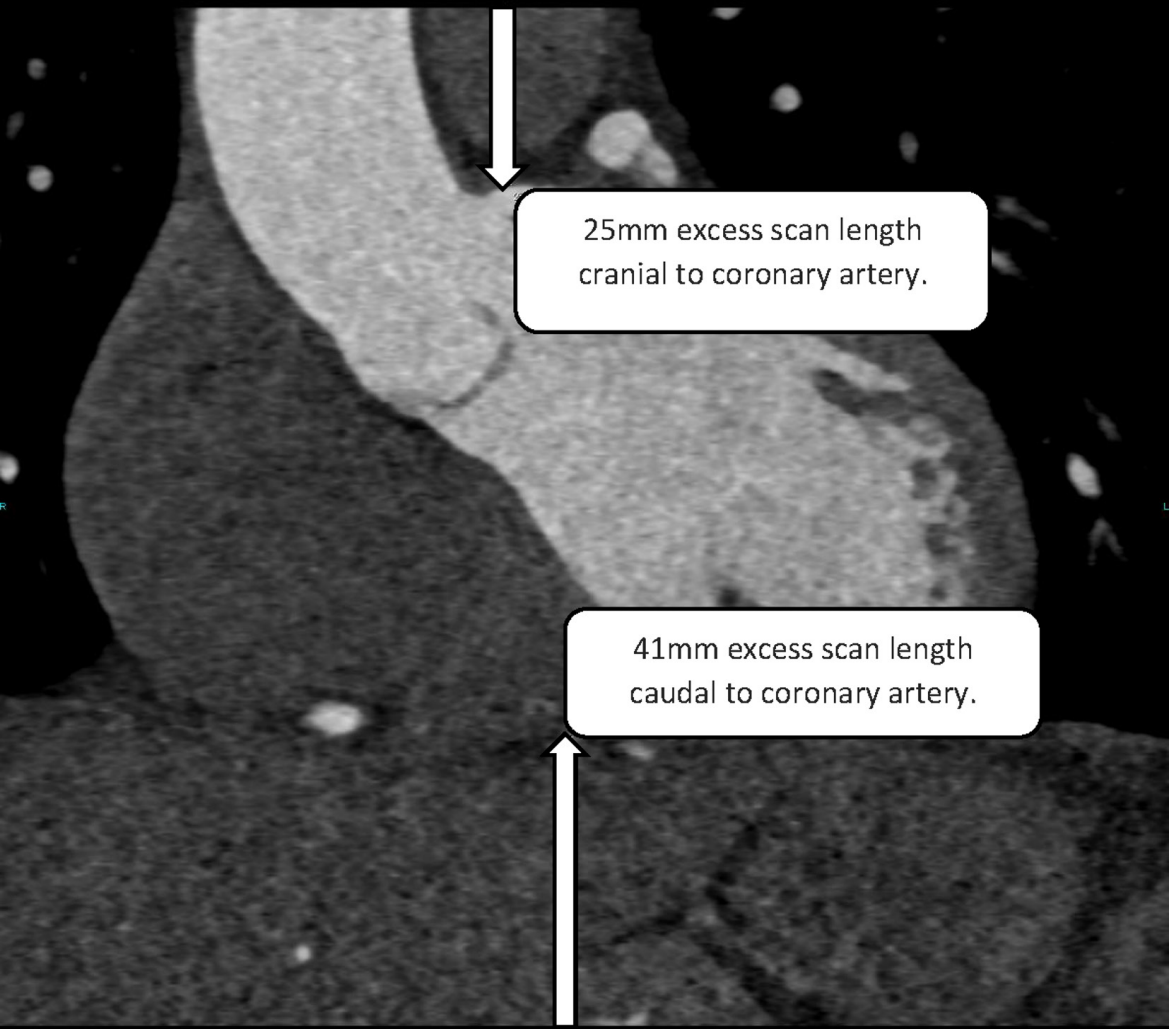
An example of measuring excess scan above (25 mm) and below (41 mm) the coronary arteries. The most superior aspect of the coronary arteries and the most inferior aspect of the coronary arteries were defined on axial images. These were cross-referenced with the coronal reformatted images shown here. The maximum distance above and below the coronary arteries is then measured. These measurements are then added to obtain the total excess scan length with a 10mm tolerance.

Ideal scan length was defined as from the most superior coronary artery to caudal to the most inferior myocardium and coronary artery, whichever was most caudal. For each patient, the superior most coronary artery was identified on the axial images, which was cross-referenced with the sagittal and coronal reconstructed images. The distance from the most superior coronary artery to the superior aspect of the scan was measured (Figure 1). The inferior most coronary artery was then identified on the axial images and cross-referenced with the sagittal and coronal reconstructions. The distance from the most inferior coronary artery to the inferior aspect of the scan was measured (Figure 1). Any measurement greater than 10 mm was defined as excess scan length, allowing a small tolerance for any differences in patient respiration between the planning scan and CTCA.^11^

### Estimation of CT effective dose

The DLP from localisers, Sn filter non-contrast planning scans (where performed), initial CTCA and any repeat CTCA due to incomplete anatomical imaging of the coronary tree (where performed) was recorded. The latter was determined from the template clinical report stating the reason for repeat imaging and consensus review in cases of uncertainty. A conversion factor of 0.026 mSvmGy^−1^cm^−1^ has been suggested for the estimation of effective dose from DLP for cardiac scans.^21^ This was applied to DLPs to provide an estimate of effective dose. Effective dose contributions from fixed-angle localisers were estimated using simulations carried out using the PCXMC dose calculator v. 2.0,^22^ similar to previously published work.^8^

### Image quality

Objective image quality was measured by measuring the CT density of the ascending aorta, left anterior descending (LAD), right coronary (RCA) and left circumflex arteries (LCx). A circular region of interest covering the maximum area was chosen. Vascular calcification was excluded from this region of interest. The attenuation value was measured where the vessel appeared largest on a single axial slice.

### Statistics

Mean, interval and *p* values were calculated for the objective measures. Student’s *t*-test was performed using Microsoft Excel (Microsoft, Redmond, WA). Inter- and intraobserver variability of overscan measurements was performed in 20 scans and calculated using an intraclass correlation coefficient (ICC) using two-way mixed, absolute agreement and average measures. Statistical significance was set at two-tailed *p* < 0.05.

## Results

### Demographics

Protocol A consisted of 47 consecutive patients and Protocol B consistent of 48 consecutive patients. There was no significant difference in age, sex, height, weight or body mass index (BMI) (Table 1).

**Table 1.** Comparison of patient demographics between Protocol A and Protocol B

	Protocol A	Protocol B	*p*-value
**Sex % male (n**)	44% (21/47)	63% (30/48)	=0.068
**Age in years (interquartile range**)	60 (48–72)	63 (50–76)	=0.233
**Body mass index kg/m** ^**2**^ (**interquartile range**)	27 (22–32)	29 (21–37)	=0.264

### Excess scan length

The inclusion of an ultra-low dose Sn-filter planning scan into the CTCA protocol was associated with a significant reduction in total scan length compared to CTCA planned using localiser alone (124 ± 13 mm *vs* 117 ± 13 mm, *p* = 0.007) (Table 2). There was a significant reduction in excess scan length caudal to the inferior aspect of the heart (excess scan length of 11 ± 8 mm *vs* 6 ± 4 mm, *p* = 0.001). There was a trend towards reduction in excess scan length cranial to the left main stem which did not reach statistical significance (excess scan length of 7 ± 6 mm *vs* 4 ± 3 mm, *p* = 0.09). Total excess scan length was significantly less in the ultra-low dose Sn-filter planning scan group (12 ± 9 mm *vs* 5 ± 4 mm, *p* = 0.001).

**Table 2.** Comparison of excess scan length cranial and caudal to coronary arteries and total excess scan length between Protocol A and Protocol B

	Protocol A	Protocol B	*p*-value
**Total scan length mm mean (interquartile range**)	124 (111–137)	117 (104–130)	=0.007
**Excess scan length > 1 cm cranial to left main stem mm mean (interquartile range**)	7 (1–13)	4 (1–7)	=0.09
**Excess scan length > 1 cm caudal to inferior cardiac border mm mean (interquartile range**)	11 (3–19)	6 (2–10)	=0.001
**Total excess scan length mm mean (interquartile range**)	12 (3–21)	5 (1–9)	=0.001
**% excess scan of total scan length mean (interquartile range**)	11 (4–18)	6 (2–10)	=0.003

### Radiation dose

Overall, there was no significant difference in the total study DLP for Protocol A *vs* Protocol B (Table 3). Using a 0.026 conversion factor applied to DLPs, and calculations of effective dose for the localisers, total study effective dose was 2.1 mSv for Protocol A and 2.2 mSv for Protocol B.

**Table 3.** Comparison of overall dose between Protocol A and Protocol B

	Protocol A	Protocol B	*p*-value
**Total study DLP^*a*^ (mGy.cm**)	mean 80, median 54	mean 82, median 46	0.44
**Total study effective dose^*b*^ (mSv**)	2.1	2.2	0.46
**Total DLP (mGy.cm), not including repeat CTCA phases**	mean 48, median 42	mean 51, median 39	0.32
**Total effective dose (mSv), not including repeat phases**	1.3	1.36	0.37
**Repeat CTCA for incomplete coronary imaging % (n**)	13 (6/47)	6 (3/48)	0.28
**Localiser DLP (mGy.cm**)	1 (SD 0.14, range 0.7–1.5)	0.5 (SD 0.11, range 0.3–0.9)	9.9
**Non-contrast tin-filter planning scan DLP (mGy.cm**)	-	3 (SD 1.54, range 1.9–10.8)	
**Test bolus DLP (mGy.cm**)	6 (SD 1.26, range 3.9–11)	6 (SD 1.39, range 3.2–11)	0.24
**Initial FLASH CTCA DLP (mGy.cm**)	43 (SD 27.4, range 10.6–115.3)	42 (SD 33, range 11.7–169.9)	0.81
**Repeat CTCA DLP (mGy.cm**)	97(SD 96.4, range 9.3–337.5)	157 (SD 159.49, range 27.7–478.2)	0.07

CTCA, CT coronary angiography; DLP, dose–length product.

a Topogram + Sn-filter planning scan where appropriate + initial FLASH + repeat for incomplete coronary imaging where appropriate.

b Using 0.026 conversion factor.

### Repeat CTCA phases

The CTCA component of the study was repeated 14/47 patients in Protocol A (30%), compared to 9/48 (19%) of patients in Protocol B. In Protocol A, six studies were repeated due to incomplete coverage of the cardiac anatomy compared to three studies in Protocol B (*p* = 0.28). In both Protocol A and B, five studies were repeated due to motion artefact. The reason for repeating the CTCA was not documented in three cases in Protocol A and one case in Protocol B. In these cases, the images were reviewed to confirm that coronary anatomical coverage was complete and not the reason for repeating the scan.

### Image quality

The objective attenuation measurements (HU) for the ascending aorta, RCA, LCx and LAD in each protocol group were measured (Table 4). There was no significant difference in the ascending aortic (*p* = 0.838), RCA (*p* = 0.263), LCx (*p* = 0.198) or LAD (*p* = 0.370) attenuation values between Protocol A and Protocol B.

**Table 4.** Objective attenuation measurements (HU) for the ascending aorta, RCA, LCx and LAD artery in each protocol group

	Vessel attenuation HU mean (IQ range)		
Vessel	Protocol A (*n* = 47)	Protocol B (*n* = 48)	*p*-Value
**Aortic root**	666 (289–1102)	674 (296–1364)	*p* = 0.838
**RCA**	612 (275–1165)	654 (225–1241)	*p* = 0.263
**Circumflex artery**	553 (263–879)	594 (312–997)	*p* = 0.198
**LAD artery**	528 (290–954)	556 (290–887)	*p* = 0.370

HU, Hounsfield unit; IQ, interquartile; LAD, left anterior descending; RCA, right circumflex artery; RCA, right coronary artery.

## Discussion

CTCA is a diagnostic imaging modality for the assessment of CAD and is recommended for assessment of stable chest pain of suspected cardiac origin.^13^ Whilst the radiation dose from radiological studies should always be as low as practicable whilst maintaining diagnostic quality, this is particularly pertinent in stable chest pain where the patient cohort is relatively young, thus with a greater theoretical risk of cancer associated with ionising radiation.^23^

Several radiation dose reduction techniques have been introduced in recent years in CTCA including prospective ECG-triggered scanning, heart rate control, reduced tube voltage and tube current modulation.^5^ This has resulted in rapid improvements in the reduction of radiation dose from CTCA; the British Society of Cardiovascular Imaging and the British Society of Cardiac Computed Tomography conducted an audit of radiation dose from coronary CT angiography in 2014 and 2016. In the 2-year interval, they identified a 30% reduction in the median exam DLP for prospective ECG-gated acquisitions with tube current padding.^24^

Optimising z-axis coverage is a simple technique to reduce radiation dose, by only scanning the anatomical region of interest whilst limiting extra scanning at the cranial and caudal ends of the study which do not add diagnostic information. One technique of optimising z-axis coverage in CTCA is to identify the relevant anatomy on a non-contrast scan performed prior to CTCA, such as a calcium score.^11^ This approach has been shown to reduce the radiation dose of the subsequent CTCA by 16%,^11^ but this comes at the cost of a relatively high dose of the overall study, adding an additional 1–3 mSv over CTCA alone.^14^

We investigated whether an ultra-low dose Sn-filter planning scan can be added to a routine CTCA protocol where z-axis coverage is planned using a single localiser. This significantly reduced z-axis overscan beyond the anatomy of interest, with no impact on image quality.

### Dose–length product

The use of third generation, prospectively ECG-triggered CT with very high pitch enables acquisition of the heart within a single cardiac cycle at lower dose (Siemens Definition FLASH, Siemens Healthcare, Forchheim, Germany). The mean DLP for the Sn-filter calcium score was 3 mGy.cm, and there was no significant difference in total study DLP following the addition of this phase to the CTCA protocol, median 54 *vs* 46 mGy.cm respectively. The dose in our audit compares favourably with a survey of CTCA practice across the UK, where the median DLP was 209 mGy.cm across 50 centres.^24^ This survey found the highest DLPs were due to use of older scanner technology. Another UK centre using high pitch FLASH mode acquisition gave median DLPs for FLASH CTCA of 59 mGy.cm,^25^ similar to the values described in the present study.

### CTCAs needing repeat phases

3/48 studies in the ultra-low dose Sn-filter planning scan group required repeat CTCA, due to incomplete anatomical coverage, compared to 6/47 in the group where CTCA was planned using a localiser only (*p* = 0.28). Inclusion of the planning scan did not eliminate need for repeats entirely, reflecting the inherent intrapatient variables such as differences in depth of breath-hold and beat-to-beat variations in heart rate and ectopic beats that may cause non-diagnostic CTCA.

The mean DLP of repeat CTCA phases in Protocol A and Protocol B was 97 and 157 mGy.cm respectively, two to three times greater than the DLP of the preceding FLASH mode CTCA sequence. When CTCAs were repeated, a higher dose protocol was invariably used, such as a step and shoot protocol using 60–80% padding. Step and shoot scanning also increases excess dose by overscan in the z-axis due to the need for ‘blocks’ of fixed length. Repeat scanning therefore significantly increases the total radiation dose to the patient by more than double. Any repeat scanning will also increase intravenous contrast load.

### Other benefits

Performing a planning scan prior to CTCA may have the additional benefit of CTCA optimisation by altering scan parameters based on the amount of coronary artery calcium present. Beam hardening in densely calcified coronary arteries causes decreased sensitivity and specificity of CTCA in patients with high coronary artery calcification.^26^ The artefact may be reduced by changing the acquisition parameters, such as kVp, padding and choice of prospective/retrospective gating.^27^

Optimising scan length also optimises the point of scan acquisition, so ECG gating is accurate when the left main stem is imaged. With a low dose planning scan, the left main stem can be identified and the scan started at an optimal position allowing for optimum temporal resolution of the proximal vessels. This is particularly important in CTCA in light of the ISCHEMIA trial, which demonstrated that patients with stable symptoms could be risk stratified using CTCA to exclude significant left main stem disease, and then managed with optimum medical treatment alone, reserving invasive coronary angiography for those with refractory symptoms.^28^

### Limitations

Our study has several limitations. The data were collected from a single centre, retrospective audit with a small sample size. It is important to note that this study was conducted as an audit, and as such the radiographers performing the examinations were not blinded to the protocol. This would have been impossible in practice as one protocol was clearly distinguishable from the other at the time of scanning. Nevertheless, scan length is an objective measure with no scope for subjectivity. A further limitation is that the rate of incomplete coronary coverage in the control group may not be representative of wider practice as scan length and coronary coverage varies between institutions.

## Conclusion

In conclusion, the present audit indicates that incorporation of an ultra-low dose unenhanced planning scan into the CTCA protocol facilitates optimal scan length for the diagnostic CTCA, significantly reducing overscanning with no significant dose penalty or impact on image quality.
